# 1044. *In vitro* Activity of Tebipenem Against Relevant Clinical Isolates in the Presence of Pulmonary Surfactant

**DOI:** 10.1093/ofid/ofab466.1238

**Published:** 2021-12-04

**Authors:** S J Ryan Arends, Abby L Klauer, Nicole Cotroneo, Ian A Critchley, Rodrigo E Mendes

**Affiliations:** 1 JMI Laboratories, North Liberty, Iowa; 2 Spero Therapeutics

## Abstract

**Background:**

Tebipenem (TBP) is an orally administered broad-spectrum carbapenem antibiotic under development for the treatment of acute pyelonephritis and complicated urinary tract infections. This study evaluated the effect of bovine pulmonary surfactant (BPS) on the *in vitro* activity of TBP and ertapenem (ETP) against a recent collection of clinical isolates.

**Methods:**

A total of 10 isolates recovered from patients with infections in 2018 were tested for antimicrobial susceptibility to TBP and ETP in the absence or presence of 1%, 5%, or 10% BPS (Infasurf; ONY Biotech). These isolates included the following species: *C. freundii*, *E. cloacae*, *E. coli*, *H. influenzae*, *H. parainfluenzae*, *K. pneumoniae*, methicillin-susceptible *S. aureus*, *M. catarrhalis*, *S. pneumoniae*, and *S. pyogenes*. Isolates were tested with the appropriate broth microdilution method for each organism as specified by CLSI. For most organisms, MICs were determined in cation-adjusted Mueller-Hinton broth (CAMHB). CAMHB was supplemented with 2.5-5% lysed horse blood for streptococci and *Haemophilus* Test Medium broth for *Haemophilus* spp. Daptomycin (DAP) was tested against *S. aureus* ATCC 29213 as a positive control.

**Results:**

All isolates displayed TBP MIC values ranging from ≤0.004 to 0.06 mg/L in media without BPS. There were no observed MIC increases >2-fold in the presence of BPS. 4 of the 10 isolates displayed slightly higher (≥4-fold) ETP than TBP MIC values. The ETP MIC values ranged from 0.015-0.25 mg/L in media without BPS. Similarly, there were no observed instances of a >2-fold shift toward lower potency in the presence of BPS. For both TBP and ETP, MIC endpoint values were easily determined, except for in the case of the 2 *Haemophilus* strains growing in the presence of 5% or 10% BPS. For these conditions, resazurin was added to establish an MIC value. The MIC values found with this method did not differ from the MIC values found in either HTM media or HTM media with 1% BPS. As expected, the addition of BPS shifted DAP *S. aureus* MIC values to >8 mg/L for all 3 BPS concentrations.

**Conclusion:**

TBP displayed potent activity against all isolates tested, as all observed MIC values were ≤0.06 mg/L. The addition of BPS to the testing medium did not affect the *in vitro* MIC values of TBP or ETP against these species.

**Disclosures:**

**S J Ryan Arends, PhD**, **AbbVie (formerly Allergan**) (Research Grant or Support)**GlaxoSmithKline, LLC** (Research Grant or Support)**Melinta Therapeutics, LLC** (Research Grant or Support)**Nabriva Therapeutics** (Research Grant or Support)**Spero Therapeutics** (Research Grant or Support) **Abby L. Klauer, n/a**, **Cidara Therapeutics, Inc.** (Research Grant or Support)**Spero Therapeutics** (Research Grant or Support) **Nicole Cotroneo**, **Spero Therapeutics** (Employee, Shareholder) **Ian A. Critchley, Ph.D.**, **Spero Therapeutics** (Employee, Shareholder) **Rodrigo E. Mendes, PhD**, **AbbVie** (Research Grant or Support)**AbbVie (formerly Allergan**) (Research Grant or Support)**Cipla Therapeutics** (Research Grant or Support)**Cipla USA Inc.** (Research Grant or Support)**ContraFect Corporation** (Research Grant or Support)**GlaxoSmithKline, LLC** (Research Grant or Support)**Melinta Therapeutics, Inc.** (Research Grant or Support)**Melinta Therapeutics, LLC** (Research Grant or Support)**Nabriva Therapeutics** (Research Grant or Support)**Pfizer, Inc.** (Research Grant or Support)**Shionogi** (Research Grant or Support)**Spero Therapeutics** (Research Grant or Support)

